# A health-systems journey towards more people-centred care: lessons from neglected tropical disease programme integration in Liberia

**DOI:** 10.1186/s12961-023-00975-x

**Published:** 2023-04-13

**Authors:** Laura Dean, Rachel Tolhurst, Gartee Nallo, Karsor Kollie, Anthony Bettee, Sally Theobald

**Affiliations:** 1 Department of International Public Health, Liverpool School of Tropical Medicine, Pembroke Place, Liverpool, L3 5QA UK; 2 University of Liberia Pacific Institute for Research and Evaluation, Monrovia, Monsterrado Liberia; 3 Neglected Tropical Disease Programme, Ministry of Health, Government of Liberia, Monrovia, Monsterrado Liberia

**Keywords:** People centred health systems, Integration, Neglected tropical disease, Health equity, Policy and programme reform

## Abstract

**Background:**

Neglected tropical diseases (NTDs) are associated with high levels of morbidity and disability as a result of stigma and social exclusion. To date, the management of NTDs has been largely biomedical. Consequently, ongoing policy and programme reform within the NTD community is demanding the development of more holistic disease management, disability and inclusion (DMDI) approaches. Simultaneously, integrated, people-centred health systems are increasingly viewed as essential to ensure the efficient, effective and sustainable attainment of Universal Health Coverage. Currently, there has been minimal consideration of the extent to which the development of holistic DMDI strategies are aligned to and can support the development of people-centred health systems. The Liberian NTD programme is at the forefront of trying to establish a more integrated, person-centred approach to the management of NTDs and provides a unique learning site for health systems decision makers to consider how shifts in vertical programme delivery can support overarching systems strengthening efforts that are designed to promote the attainment of health equity.

**Methods:**

We use a qualitative case study approach to explore how policy and programme reform of the NTD programme in Liberia supports systems change to enable the development of integrated people-centred services.

**Results:**

A cumulation of factors, catalysed by the shock to the health system presented by the Ebola epidemic, created a window of opportunity for policy change. However, programmatic change aimed at achieving person-centred practice was more challenging. Deep reliance on donor funding for health service delivery in Liberia limits the availability of flexible funding, and the ongoing funding prioritization towards specific disease conditions limits flexibility in health systems design that can shape more person-centred care.

**Conclusion:**

Sheikh et al.’s four key aspects of people centred health systems, that is, (1) putting peoples voices and needs first; (2) people centredness in service delivery; (3) relationships matter: health systems as social institutions; and (4) values drive people centred health systems, enable the illumination of varying push and pull factors that can facilitate or hinder the alignment of DMDI interventions with the development of people-centred health systems to support disease programme integration and the attainment of health equity.

## Background

Neglected tropical diseases (NTDs) are associated with mortality and high levels of morbidity and disability as a result of stigma and social exclusion [1–4]. The WHO 2020 road map, ‘accelerating work to overcome the global impact of neglected tropical diseases’, prioritized the control, elimination and in some cases eradication of these diseases by 2020, through two major strategies [5]. The first strategy was innovative and intensified disease management (IDM), which supports disease management through the primary health care system. The second focused on preventive chemotherapy and transmission control (PCT) through the implementation of large-scale, population-based drug administration, usually termed mass drug administration (MDA) [5]. MDA originated from the African Programme for Onchocerciasis Control (APOC), whereby freely donated medicines were distributed by community health volunteers to at-risk populations in response to the high levels of visible suffering resulting from river blindness [6]. Despite an early focus on the alleviation of suffering, it is the people affected by NTDs who have arguably become the most forgotten throughout multiple decades of vertical NTD programme delivery focused on MDA. Furthermore, both IDM and PCT have commonly had a heavy biomedical focus, with limited acknowledgement of the social causes and consequences of diseases [4]. Given that the majority of these NTDs do not cause death but instead lifelong morbidity and disability, a more holistic approach to the management of NTDs is needed that supports affected persons to negotiate the physical, psychological and social implications [2, 4]. WHO’s new NTD roadmap (2021–2030) ‘ending the neglect to attain the sustainable development goals’ recognizes the need for revived cross-cutting action to provide integrated people-centred care for persons affected by NTDs [7]. However, the evidence base that can support the development of integrated person-centred approaches to NTD management is still emerging.

People-centred health systems (PCHS) are viewed as essential by many in the health systems community to ensure the efficient, effective and sustainable attainment of universal health coverage (UHC) [8–10]. An essential value in the development of PCHS is a movement away from a system focused on health institutions or disease, to one that focuses on the needs of people, whilst recognizing the central importance of relationships and values in driving systems change [11, 12]. Thus, PCHS favour integration of vertical disease programmes that enables:

*‘health services to take the responsibility to operate specific activities designed to control a health problem…and become one of several channels for the programme to implement its activities, which then become part of the broader package of activities delivered by these multipurpose general health services (13pA2)’*

However, within health systems discourses, the relationship between disease control programmes and health services, and the added value of disease control programme integration, has long been debated [13, 14]. Tensions are perceived due to a dichotomy in the underlying value base or objective of disease programmes in comparison with those of integrated and generalized health systems [13–15]. Criel et al. [13] and Marchal et al. [14] present comparisons of the main elements of disease control and health systems perspectives as shown in Table 1.

**Table 1:** Core elements underpinning disease control and health systems perspectives

	Disease control programmes	Generalized health care systems
Objective	Reduction of burden of disease	Contribute to physical, mental and social wellbeing
Analysis of health problem	Focus on the presence of disease in population	Focus on people
Decision-making criteria	Evidence of burden of disease and cost-effectiveness	Technical, social and political criteria
Strategic approach to implementation	Short-term actions based on technical solutions and aiming at rapid results	Long-term iterative approach aims at protecting people and responding to needs
Concept of ‘community’	Intervention target, beneficiaries	Beneficiaries and drivers to which health services are accountable
Concept of ‘participation’	Target orientated: needed to fulfil goals	Empowering

Source: Adapted from [13, 14]

The development of PCHS aligns to the priorities of integrated and generalized health systems. PCHS are often most successful when linked to other efforts or drivers for change, for example in improving health equity [16], as PCHS demand shifts in accountability away from compliance to government-defined targets (bureaucratic accountability) towards systems that enable responsiveness to the needs of service users (external social accountability) [17, 18]. Health systems become empowering, and users become the stakeholders to whom services are accountable [13]. Quality of life—as opposed to quality of care—becomes the critical foci of system design, which is driven by holistic needs of communities and social health determinants rather than common epidemiological profiles [8]. Co-production of services between communities, providers and policy makers is prioritized, supporting a shift from paternalistic care delivery towards enabling systems strengthening and ultimately shaping improved health and wellbeing [8, 9].

**Fig. 1:**
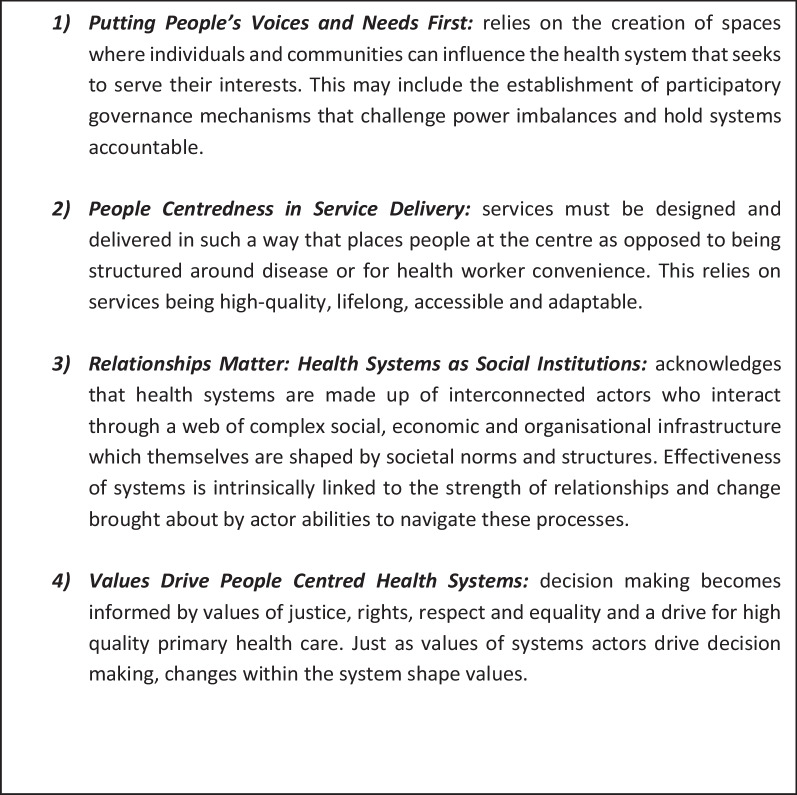
Summary of Sheikh, Ranson and Gilson (2014) core aspects of people-centred health systems

Sheikh, Ranson and Gilson [12] draw this thinking together and outline four core aspects that are central to the development of integrated PCHS: (1) putting people’s voices and needs first; (2) emphasizing people centredness in service delivery; (3) viewing health systems as social institutions; and (4) understanding that values drive people centred health systems (Fig. 1) [12]. To date, a focus on biomedical management of NTDs, driven by the priorities of large pharmaceutical corporations and international donors [19], has dominated academic literature, policy and programming related to NTDs, arguably in opposition to integrated people-centred approaches. Terms such as morbidity management and disability prevention (MMDP) that focus on the cure, prevention or medical management of disease condition reflect this and have translated to little importance being placed on the lived experiences and values of affected populations [3, 4], thus compromising the delivery of services that place people at the centre.

**Fig. 2:**
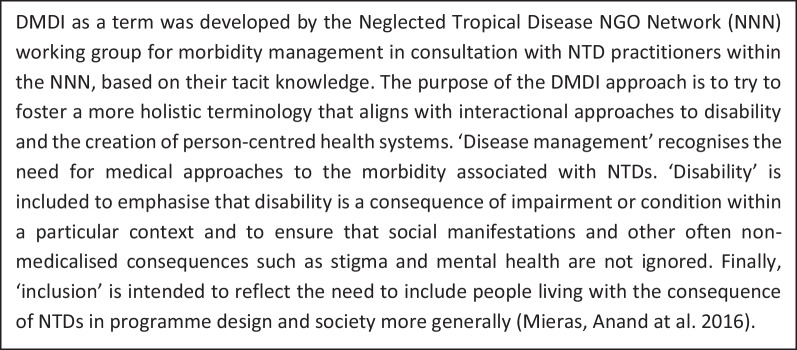
The origins of disease management, disability and inclusion for neglected tropical diseases

However, as reflected within WHO’s 2021–2030 NTD roadmap, there is a shift in thinking amongst NTD practitioners and associated policy dialogues to focus on strategies that promote the more holistic concept of disease management, disability and inclusion (DMDI) as further described in Fig. 2 [4]. The prioritization of DMDI is aimed at ensuring a full integrated person-centred continuum of care for individuals affected by NTDs, rather than one which is dominated by biomedical approaches and marginalizes lived experiences [4].

Criel et al. [13] suggest that decisions about what and how to integrate disease control programmes are complex. Firstly, it is necessary to understand how desirable integration is, i.e. what is the added value in asking health care systems to add disease-focused activities into routine service provision? Secondly, is integration possible based on ability to standardize tasks and the necessity of specialized services? Thirdly, what is the opportunity of integration, i.e. can it help or hinder health systems development [13]? Despite integration being perceived as desirable for DMDI service delivery, little is currently known about how to effectively shift from NTD programmes that run parallel to routine health service delivery and focus on MDA, to those which focus on integrated, people-centred, longitudinal and lifelong care. Furthermore, in the development of more integrated, people-centred approaches in the response to NTDs, there is currently minimal synthesis of evidence from the broader health systems literature regarding factors that promote such approaches. The aim of this paper is to explore the opportunity and possibility that the development of integrated DMDI strategies for NTDs present for the development of PCHS and to articulate how this learning can support the attainment of health equity for affected populations.

Literature on health systems strengthening emphasizes that systems are highly context dependent and shaped by complex social dynamics [12]. It is critical to be able to understand and address such dynamics in the development and implementation of new interventions, as it is these complex and locally constituted relationships that shape how different processes of systems integration occur [11]. Current narratives surrounding the development of integrated, people-centred responses to NTDs are largely framed within global rather than local terms, which is potentially problematic in supporting the development of context-specific solutions that are responsive to locally constituted relationships [11].

The Liberian NTD programme is at the forefront of trying to establish a more integrated, person-centred approach to the management of NTDs through the development of their ‘Integrated Case Management Strategy’ [20]. This strategy focuses on DMDI for a number of endemic NTDs and their associated morbidities including, Buruli ulcer, lymphoedema, hydrocele, leprosy and Yaws [20]. The four core pillars of integration within this approach were: (1) government ownership and partnership across divisions to enhance resource management; (2) resource mobilization by integrating NTDs within all relevant national policies and increasing community awareness; (3) scaling up access to interventions with a specific focus on active case searching through MDA campaigns, establishing a centre of excellence for NTDs, strengthening the supply chain, and ensuring better community access to treatment and management interventions; and (4) improving surveillance through integration of NTDs within data management systems. Prior to the development and launch of this plan in October 2016, there was no clear DMDI strategy; disease management associated with NTDs was completed on an ad hoc basis [1]. The context of programme and policy reform in Liberia is therefore used as a case study within this paper. Drawing on the varying experiences of national programme implementers and non-governmental development organizations (NGDO) partners, we explore the creation and roll-out of a national integrated DMDI policy for NTDs. We consider how far the key aspects of NTD programme reform align to the discourse around the development of PCHS and to what extent social relationships influence the successes and failings within the process.

## Methods

### Study design

**Fig. 3:**
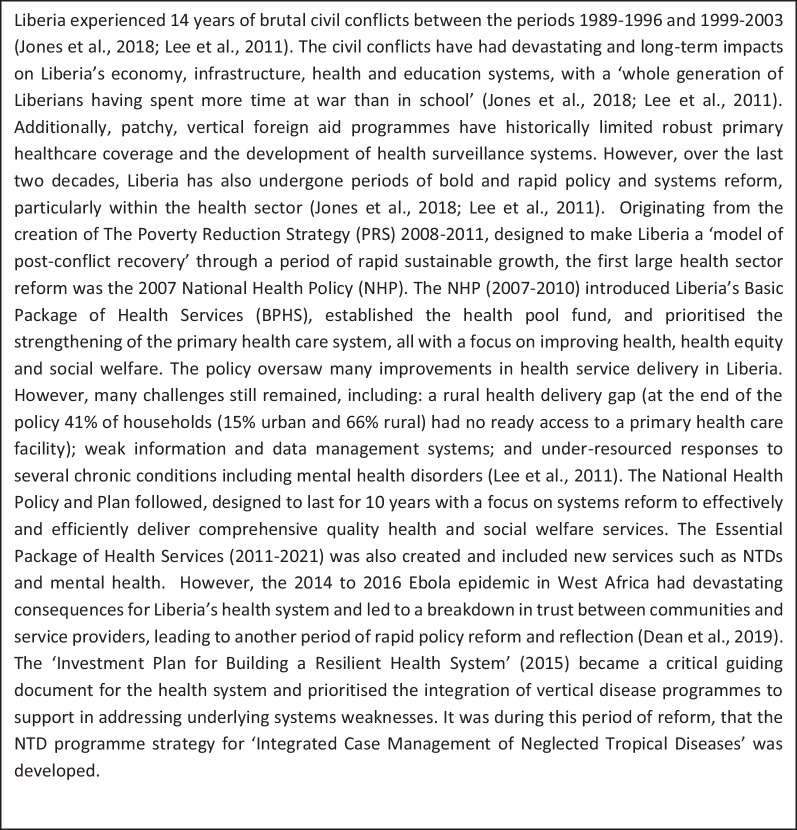
Liberia—the case study context

We use a qualitative case study approach to explore how policy and programmatic reform of a vertical NTD programme supports systems change towards the development of people-centred systems and services. Stake [21] describes our single case study approach as ‘instrumental’ as it is designed to facilitate thinking within NTD and health systems communities [21] regarding the specific issue of DMDI service integration and the development of PCHS, as opposed to being thought of as ‘typical of other cases’ [21, 22]. Our case study approach allows for the intense focus on a single phenomenon (policy and programme reform) within a real-life context (Liberia—see Fig. 3). Through the use of multiple data sources, our exploration acknowledges that ‘cases’ and contexts are constantly changing and multiple variables and considerations bring complexity to our analyses [22, 23].

### Data collection

Data collection took place between December 2016 and December 2018 and involved interviews with key informants and ethnographic observations of meetings at national and international level. Data were collected by L.D. (MSc) and G.N. (MSc), both of whom have experience collecting data with stakeholders across all levels of the health system.

#### Key informant interviews

We conducted 13 individual and 1 paired semi-structured interview(s) with purposively selected key informants at the national and county level. Key informants were selected due to their role in NTD programme delivery or associated activities and included: Civil Society organizations, NGDOs or donor representatives [4]; National Ministry of Health staff [6] and members of the county health team [4] from three counties where integrated case management activities are currently being implemented (Bong, Nimba and Maryland). Interviews explored the generation and content of the integrated case management plan; implementation of integrated disease management; and informants’ perceptions of key strengths and challenges for disease management, disability and inclusion.

### Data analysis

We recorded all interviews and transcribed them verbatim. Data were stored and analysed using NVIVO 10. Notes from participant observations were also typed up and, where required, points of clarity discussed with G.N. (local field assistant) and the NTD programme team. We analysed all data thematically. Initially we coded grouped data inductively to explore core factors that were related to the interface between NTD programmes and the health system in relation to (a) policy development and (b) policy or programme implementation. Subsequently, higher-level analysis was guided by Sheikh, Ranson and Gilson [12] core aspects of people-centred health systems (Fig. 1). Data analysis was completed as a collaborative process between L.D., R.T., G.N. and S.T.; K.K. and A.B. were consulted to support with data interpretation.

### Reflexive diary

To enhance the trustworthiness of key informant interview analysis, this manuscript also draws on experiences of the lead author (L.D.) as documented in a reflexive diary. This included critical reflections from key meetings, discussions and county supervision activities that were relevant to the development, adaptation and implementation of the integrated case management strategy. Detailed field notes and critical reflections were taken throughout the data collection period.

## Results

Our results are organized into three key sections with emergent themes linked to each subsection also presented. The first theme, policy development, focuses specifically on NTD policy reform in Liberia in the wake of the Ebola epidemic. The second theme, policy and programme implementation, is concerned with how policy change translates to change within the NTD programme. Finally, theme 3, reflections and the road ahead, explores challenges and the way forward for the NTD programme in Liberia as it aims to development more person-centred responses to NTDs.

### Policy development

#### Maximizing a window of opportunity for policy and programme change

The creation of the integrated case management plan in Liberia was shaped by the cumulation of multiple factors that created a clear window of opportunity for policy and programme change. Informants described how integration of various disease programmes, specifically leprosy and Buruli ulcer, had been a key national NTD programme priority for many years with the co-implementation, primarily of disease mapping activities, beginning just prior to the Ebola outbreak. Ebola interrupted the progression of such activities and limited the establishment of a fully integrated NTD programme whilst also emphasizing clear health systems weaknesses.

In the period immediately after the Ebola outbreak, health systems priorities were observed to change, with a range of actors within the health system coming together to work out the best way forward to be more responsive and resilient to the population’s health needs. National health policy reform, including the establishment of the ‘Investment Plan for Building a Resilient Health System in Liberia’ prioritized a push towards programme integration [24]. The merging of programmes, particularly those focused on NTDs requiring case management, was therefore seen as essential ‘to save resources and time’ (National MoH Staff). It was during this time that the NTD programme was able to use adjustments within national policy that prioritized a shift towards vertical programme integration to lobby support and political will from WHO, NGDO partners and the Ministry of Health to make a critical change to NTD policy and programme implementation structures.

*‘prior to Ebola, the voices of the Ministry of Health were absent in designing programmes. No funding was available for case management and so the MoH had very little say in policy and programme design. Following Ebola, we were motivated by access and trying to improve access to case management through the health system…we wanted to move away from a disease specific focus and reduce inequities…with vertical disease programmes, for example for leprosy, some people can access everything…whereas others can’t access anything. It seemed like a skewed way of providing development aid’ (NGDO Partner Representative)*

NGDO partners who were engaged in the process also described the need to prioritize the viewpoint of Liberian NTD programme staff and other health systems actors with a key focus of policy reform on ‘capacity strengthening of the system’ and a hope that the development of the new strategy would ‘minimise the disease focus of NTD programmes and emphasise people’ (NGDO Partner Representative). Informants emphasized the importance of ‘designing the integrated case management plan around the four pillars of the existing NTD master plan to encourage support [for programme and policy reform] from WHO’ (National MoH Staff) as it was a policy or programme format with which they were familiar.

#### Prioritizing the view of affected persons

Programme implementers from all levels of the health system were described as key in shaping the way that the integrated case management policy was designed, developed and implemented. However, no consensus was reached on the engagement of involving persons affected by NTDs in programme design and review meetings, and they were therefore excluded.

Despite this, it was apparent from interactions with multiple programme implementers that care for the improved health and wellbeing of people affected by NTDs was at the forefront of their efforts and decision making. For example, we observed that some programme implementers would pay from their own pockets for surgical costs, school fees, food and transportation of affected persons. Reflections from key informants also emphasized a desire for a change in focus away from the biomedical construction of disease and associated interventions towards more holistic responses that aligned to their value base and experiences. For example, many informants described feeling like they needed to expand service delivery to include the provision of psycho-social support. However, they felt restricted to be able to do this within the parameters of a fragile health system when they had limited evidence of what would work and where they should target resources. Many described that day-to-day interaction with affected persons made decisions about what should or should not be included within integrated programme delivery more challenging. Implementers felt compromised in their attempts to establish an integrated programme that worked, whilst still understanding the broader needs of people affected as addressing everything at once felt ‘too big’.

*‘psycho-social elements aren’t included at the moment…but it becomes a real wrestle. One reason why integrated case management is not being implemented and adopted by the NTD community is because it feels so big…livelihoods…psycho-social support…we lose the person at the beginning who really just wants to give the pill for these diseases…we had to have an element of compromise…think through what can we do that will have the best impact…’ (National MoH Staff).*

*‘I was called to go and confirm whether this client is a confirm lymphoedema. And when I got there a young girl 26 years got this lymphoedema leg and I talk to her it was confirmed that lymphoedema. We taught her to take care of the lymphoedema leg…You wouldn’t believe this girl was bold to express her heart that she is too young to live with condition…she said she is going to take her life. Right there I realized that mental complication it has…I immediately came and I went through the mental health department and I say look I got a case you guys have to get involve this is the situation, this is a declaration’ (National MoH Staff).*

Consequently, long discussions with programme staff often revealed personal distress based on multiple interactions with affected persons who they felt they could not support adequately.

*‘Because you will see somebody just sitting…depression…you will become depressed…I notice some of them may even want to commit suicide if you don’t have a good family background to talk to you…talking to you they will [shy] away from you, we need support for that’ (County MoH Staff).*

### Policy and programme implementation: as strong as the system you represent

All were committed to case management being ‘part of the regular health service delivery system of the country’ (National MoH Staff) and described seeking to maximize avenues for integration, which was often highlighted as easier at lower levels of the health system. Informants described some parts of integrated implementation working well, whereas others were seen to be limited. Many emphasized that earlier case detection by community health assistants was working particularly well, due to integrated training, supervision and motivation processes that were aligned to the community health division’s policy and programme delivery [25].

*‘unlike the past where we used to go out to actively find cases…There is a curriculum formulated to train…community health assistants, and we train the community health surveillance supervisor which is CHSS in all medical related cases. The curriculum was developed by the community health department and the NTD department’ (National MoH Staff).*

This was thought to be further enhanced by the programme’s ability to fulfil a motivation gap for some community health volunteers through the introduction of the active case search incentive policy, whereby community health volunteers (those not currently formally incentivized by the health system) receive 5 US dollars per target NTD case identified and confirmed. This strategy was designed to reduce the demotivation of community health volunteers who are not part of the national community health assistant (CHA) programme that provides 70 US dollars per month motivation to CHAs who have undergone a 4-month training programme [25].

*‘We have introduced another method called active case search incentive base and it is really for the community health volunteers…from our experience some of them were left out of the community health assistant programme so they are like demotivated and you don’t find the community health assistants in all of the communities…So we communicated with the focal points and told them to inform the community health division that every case confirm gets 5 dollars (National MoH Staff)’.*

Operational integration at the county level was also described as having been relatively straightforward, two-monthly supportive supervision visits from national programme staff to the county level every had enabled the addition of NTDs as an agenda item within weekly county medical meetings. However, it was observed that this process was smoother in some counties than others, often dependent on the capacity of the county level NTD focal point and the personal relationship between this post and the national NTD team. Integration of the multiple indicators necessary for the effective inclusion of various NTDs within health monitoring and information systems was described as being a more laborious but essential process to enable the NTD programme to become part of routine county planning activities. Additionally, one of the biggest challenges in establishing integrated service delivery, was described as the supply chain, with many implementers asking ‘how do you put something into something that is already broken?’ (National MoH Staff). This was particularly problematic for many programme implementers who found it challenging that ‘we are creating the demand and we don’t have the drugs. We don’t have the medical supply. So, in order to mitigate that we must have the drugs’ (National MoH Staff).

*‘The challenges are that some of them are lacking of this support, lacking of drugs, sometimes the drugs are not on time… counties don’t have the capacity to procure easily, so its national, national should be able to purchase more drugs’ (County MoH Staff).*

### Reflections and the road ahead: challenging deep routed verticalization, disease silos and donor control

Despite being presented with a clear window of opportunity for change in policy, programmatic change aimed at achieving person-centred practice was viewed as more challenging. Decisions about which diseases to include as part of integrated case management approaches appeared to be based on a bio-medical view of disease condition and the historic dichotomy between PCT and IDM diseases, with specific focus on addressing ‘reversible’ NTD-associated morbidity. However, over the duration of the study this viewpoint began to shift, with programme implementers becoming increasingly reflective about the inclusion of additional disease conditions and the need to link with other sectors to address wider support needs of affected persons.

*‘I want to believe initially we are looking at conditions that are with a burden, onchocerciasis is one of the burden conditions, but we focused on conditions that we could respond to and bring relief to the client. Like for onchocerciasis once you have gone blind it is difficult like that particular condition. For example, with hydrocele you can do the correction. With Buruli ulcer it can be some level of correction. With lymphoedema stage one if it is diagnosed early you can interrupt the progression. But when you diagnose at a later stage definitely you cannot help the situation. It is just to give some home base health care and health education. I want to believe that in the nearby future onchocerciasis will be included because it causes disadvantages once you cannot see’ (National MoH Staff)*

Funding of integrated approaches was, however, the most critical barrier to effective implementation of integrated programme delivery. The funding problem was described as two-fold: (1) the long-term and deep reliance on donor funding for health service delivery by the government of Liberia limits the availability of flexible funding, and (2) there is ongoing funding prioritization of donors and NGDO partners towards specific disease conditions. Informants described the resultant precarious nature of integrated programme delivery and the additional workload, stress and ongoing negotiation that such structures enforced on the national programme team was frequently observed. Many described that a change to funding flows and partnership approaches was essential to allow for the sustainability of integrated approaches.

*‘The challenges there is that we have only one partner that is actually supporting and limited funding from government to support these programmes…so most of the funding that come is through the partner so those are the challenges that are actually face with the program’ (County MoH Staff)*

Some NGDO partners described that there is limited opportunity for national NTD programmes to provide feedback to international donors regarding the rigidity of funding flows and their associated impacts on programme responses. This limits the ability of NGDO partners to work with programmes in a way that is mutually responsive to national priorities and can compromise the development of equitable partnerships between NGDO partners and national programmes. Furthermore, where NGDO partners are unwilling or unable to collaborate effectively to facilitate integrated approaches and move outside of disease silos, this was observed to be likely to limit progression towards integrated service delivery.

*‘the way things are structured internationally is the biggest reason these programmes have been implemented vertically for such a long time…Funding can be a problem, disease focused funding can be the most frustrating thing…it doesn’t really focus on the human…health workers end up having to go and do one thing for five days and then another thing for the next five days, just because we [NGDO partners] aren’t willing to work together…there is such a missed opportunity for people to work together. NTD programmes should be given more opportunities to report back…there is a lack of reflection by partners on the impact their own goals and priorities have…could NTD programmes come together to present a framework for good partnership agreement…we shouldn’t be ignorant to the fact that there is a co-dependency between NTD programmes and donors…Getting people to leave their disease silos is challenging…I don’t know how many more generations of leprosy experts we will need…but hopefully not that many…you win some you lose some…some people are open to new approaches…others are not’ (NGDO Partner).*

## Discussion

**Fig. 4:**
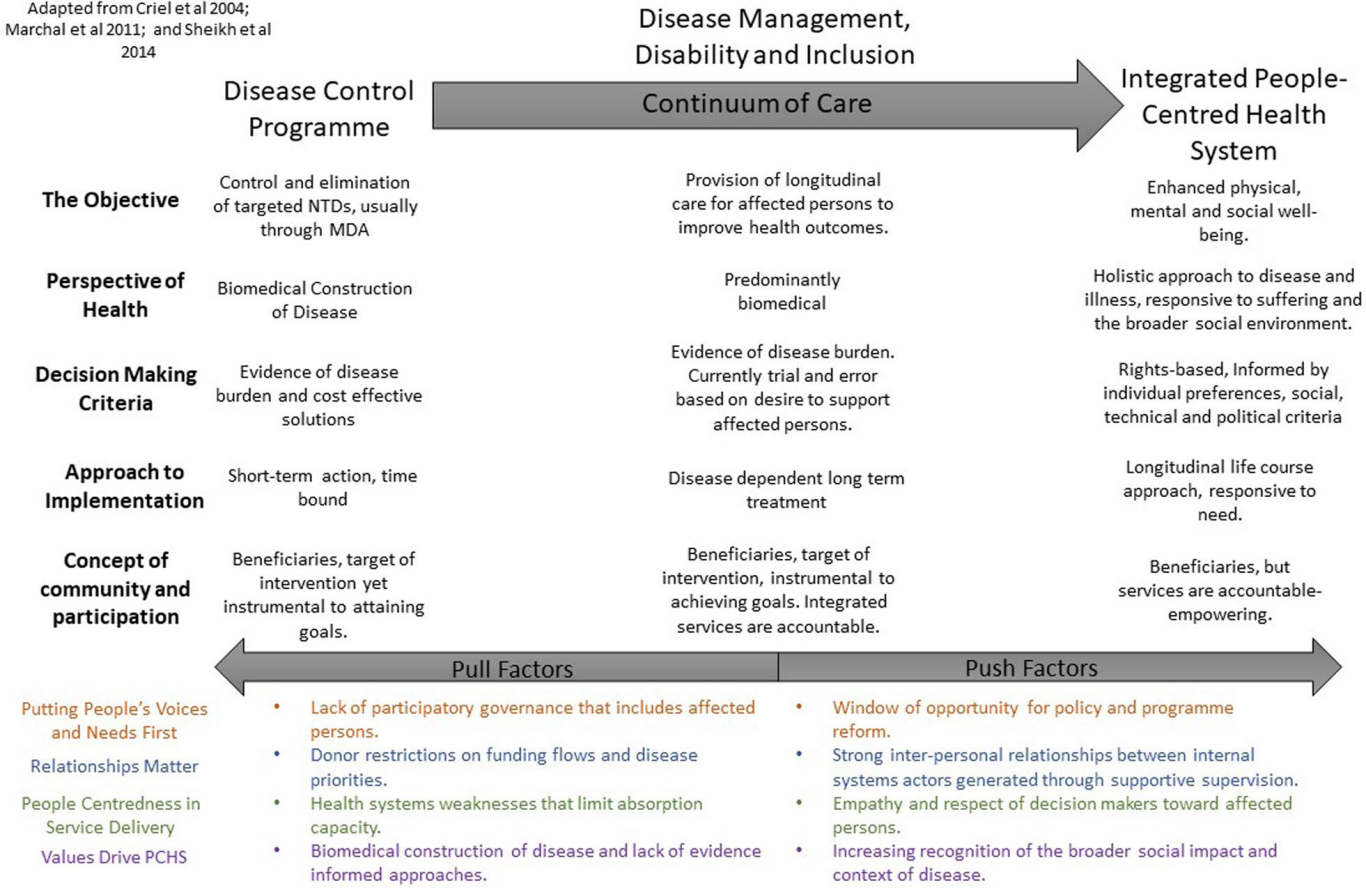
Disease management, disability, and inclusion as a bridge in supporting a transition from disease control programmes to integrated, people-centred health systems

This article aims to consider the extent to which NTD policy and programme reform can contribute towards the development of integrated PCHS through the alignment of value bases and core principles. The findings above have explored the interface between the health system and NTD policy and programme reform in relation to DMDI in Liberia. Drawing on the core elements of disease control programmes and integrated generalized health systems presented in Table 1 and adapting them to be of relevance to NTD programming in Liberia, we suggest that DMDI serves as a bridge between NTD programmes conceptualized around disease control and the development of more integrated PCHS [14, 15] (Fig. 4). Our findings illuminate multiple push and pull factors that can facilitate or hinder the alignment of DMDI interventions to the development of PCHS. Within our discussion we consider these push and pull factors in relation to Sheikh, Ranson and Gilson [12] four key aspects of PCHS (Fig. 1).

### Putting people’s voices and needs first

A central tenant of putting people’s voices and needs first is the way in which health systems are governed [12, 17]. Effective approaches to systems governance require consideration of the roles and relations of all systems actors including international NGDO partners and affected persons, not just national governments [17]. Multiple accountability relationships exist within NTD programme governance (international NGDOs and donors to national government; and national government to affected people) that need to adapt to promote the development of person-centred approaches, these relationships are discussed in turn in this subsection.

Within our study, we found that the ‘window of opportunity’ or ‘push’ for the development of an integrated PCHS in Liberia presented a critical moment for national actors to hold international NGDO partners more accountable to the provision of an NTD service that responded to their needs and priorities. This saw a long-awaited shift in the core programme objective of the Liberian NTD programme away from a sole focus on the control and elimination of NTDs, to an equally important focus on disease and health systems integration for the provision of longitudinal care for affected populations, a core value of people-centred services [26]. Thus, following a moment of health system crisis, the Liberian NTD team was able to carefully navigate deep routed and historical bureaucratic accountability of the national system (towards international targets and priorities due to chronic aid dependency [27]), and shape the redirection of their programme towards more person-centred approaches. Capacity-strengthening activities that enable a clear role and function of national actors in health governance and priority setting have been described as essential in establishing PCHS [12, 28]. Our study findings support this, and highlights the need for a key moment of reflection for the NTD community as we strive to establish person-centred approaches to DMDI. We must consider how to support the full and equitable participation of national systems actors in international agenda setting, and support the adaptation of international agendas to local contexts.

Despite these achievements at the national level, at lower levels of the health system true participation in decision making by affected persons represented a ‘pull’ for NTD programme implementers towards disease-control-centric approaches that see beneficiaries as the (passive) target of health interventions [14]. Participatory governance is essential within PCHS [17] to improve equity and ensure that those with the greatest health needs have the best ability to be able to direct resources [12], and there is increasing recognition of the capability of beneficiaries to contribute towards effective priority setting and governance processes [17, 29]. By failing to incorporate mechanisms for these contributions, the NTD programme limits advancement towards a person-centred focus. The use of patient advocates to support the participation of affected persons in priority setting and resource mobilization is increasingly prioritized within the NTD community through networks such as NTD Non-Governmental Organisation Network (NNN) [3, 30, 31]. However, our results suggest that a critical challenge remains as to ensure that these actors are given a seat at the table in national policy and programme reform. Limited active engagement of affected persons at national and subnational levels perpetuate paternalistic approaches to care delivery [9], which can hinder quality care experiences and associated quality of life for individuals and communities [32]. Supporting the health system to understand the problems of people affected by NTDs from their own vantage point is a key and critical step in supporting health practitioners and policy implementers to design strategies that enable the delivery of high-quality care [32].

### Relationships matter: health systems as social institutions

As is described in the PCHS literature [33], our findings emphasize that the role of trust and ability of national programme staff to manage relationships with external (NGDO partners) and internal health systems actors was critical in shaping how far systems could respond and adapt. For example, interpersonal relationships mattered at implementation levels of the health system where integration of service delivery seemed most permissible. Supportive supervision that established effective working relationships with county health teams enabled national actors to be responsive to the priorities of staff who are the backbone of NTD service delivery; this was seen to be an essential factor in supporting a ‘push’ towards the development of PCHS [34]. However, regardless of the strengths of these relationships and the ability of programme actors to lobby political will and shape the generation of a new NTD programme vision in Liberia, restrictions within NGDO funding flows were still observed to stall integrated programme delivery. Donor restrictions currently render some partners unable to move outside of disease specific funding silos, thus reinforcing a ‘pull’ towards disease- or issue-centric service delivery [14, 35]. This limits the responsiveness of NGDO partners and programmes to national health systems priorities and stalls the proposed paradigm shift within the NTD community towards more person-centred approaches. Furthermore, rigid funding flows can limit the ability of national programme implementers to fulfil their leadership and innovation potential as they are held accountable to the implementation parameters of international organizations who are frequently governed by a one-size-fits-all approach [36].

### People centredness in service delivery

Chronic programme verticalization, which has led to the establishment of parallel NTD programmes, shaped by the priorities of international disease experts and funding bodies [14, 35], contributed to multiple ‘pull’ factors which limit the ability of service delivery to become fully people-centred. Health systems strengthening has seldom been prioritized by the NTD community on the basis of the rationale that NTD programmes reach areas where there have been previous health systems failings and so reliance on community resourcefulness is essential [14]. Parallel provision of NTD services has therefore failed to support and address systems weaknesses, thus limiting the absorptive capacity of an already overburdened health system [14]. For example, as is the case in Liberia, weak supply chains and scarce human resources often render systems unable to respond to the needs of affected persons at primary or secondary level due to an absence of medicines and psycho-social support services. Immediate and longitudinal support becomes compromised and the provision of continuous support for affected persons difficult [32].

Engagement with community health structures is essential to improve interconnectedness between service users and providers and is critically important for improving external accountability of the health system [18]. However, it is important to reflect on how this engagement may contribute to or undermine the people centredness of service delivery. Incentivization of health workers based on disease case finding reinforces the important surveillance element of their role but can be seen as at odds with ensuring longitudinal person-centred care [26]. Furthermore, when effectiveness is measured on the basis of disease identification count, equity of service delivery can become compromised and/or distorted [32]. Thus, a critical dilemma for any vertical disease programme hoping to support the strengthening of PCHS is how best to support and motivate community health volunteers when they are not adequately or equitably supported within the generalized health system. Establishing quality measures for performance-based financing within DMDI programmes that extend beyond case detection could support in the development of a more comprehensive service [32]. Community-based comprehensive services should also seek to move beyond patient- or disease-centred interactions towards approaches that see the person as a whole [32]. Programme implementers undoubtedly evidenced empathy towards the holistic needs of affected persons as a key ‘push’ factor towards people-centred approaches. However, this is likely to be an ongoing and key test for the NTD community as a shift in focus away from disease challenges their unit of identity.

### Values drive people-centred health systems

Perhaps the strongest principle within the development of PCHS is that values are critical and important drivers within health systems reform [12]. Justice and a focus on people—not diseases—are a key principle underlying the proposed paradigm shift within the NTD community [4] and the main reason sighted for the increased inclusion of DMDI within the NTD 2021–2030 roadmap. Care for and a desire to support people affected by NTDs were unquestionably at the centre of key informants’ motivation for the case management strategies development in Liberia and represent a key ‘push’ factor towards person-centred response. However, we found that NTD programme delivery in Liberia is still orientated or ‘pulled’ towards diseases and patients. In making a shift towards the development of integrated person-centred services, a key and ongoing challenge for the NTD community emerges in terms of adjustment from bio-medical constructs of disease prevention, diagnosis and treatment to consider the holistic needs of affected persons and their families. Our study showed increasing recognition amongst programme implementers of the broader social impacts of NTDs, specifically in relation to mental ill health. However, the challenges implementers faced in having the resources or knowledge to respond emphasizes that there is a need for further evidence generation on how to make best use of scarce resources to support in systems strengthening whilst meeting the holistic needs of affected persons.

### Study limitations

We have only considered policy and programme reform from the perspective of key decision makers at national and county level within this paper. Engagement with stakeholders at lower systems levels, for example, facility staff and community health workers, who are often at the interface of implementing such reforms, would have provided useful and additional critical insights and should be considered as an area of future research. We have not explored the perspectives of people affected by NTDs in this manuscript; however, these insights are essential, and are prioritized within other publications from the same study [1]. Reflections on this process from other countries may have further supported the generalizability of these findings. However, we used a case study methodology as an instrument to facilitate thinking within the NTD and health systems community, rather than arguing that Liberia’s situation is representative of all countries embarking on the development of more integrated people-centred DMDI service delivery. Nevertheless, the processes that are in operation in Liberia have the potential to provide learning to other settings that are embarking on the delivery of the WHO’s 2021–2030 NTD roadmap and the mainstreaming of NTD services. Our findings showcase that, by prioritizing the development of strong interpersonal relationships across levels and teams within the health system; valuing the needs and priorities of affected persons through their inclusion in health governance; recognizing the broader social impact of NTDs; and maximizing opportunities for policy change, shifts towards more integrated approaches to the management of NTDs provide great opportunity for the development of more person-centred services. However, for country efforts to be successful, this must be accompanied by donor flexibility and responsiveness to the realities of those at the forefront of service delivery.

## Conclusion

The case of Liberia illustrates the opportunities and challenges in implementing a policy and programme shift towards integrated PCHS within NTD programme reform. Assessing policy and practice against Sheikh, Ranson and Gilson [12] core pillars of PCHS should be considered by the NTD community as they seek to contribute to the development of PCHS through the provision of integrated DMDI interventions.

## Data Availability

The datasets used and/or analysed during the current study are available from the corresponding author on reasonable request. All data necessary to support the analysis presented here are included in the published article.

## Abbreviations

NTDs: Neglected tropical disease
PCHS: People-centred health systems
DMDI: Disease management, disability and inclusion
PCT: Preventive chemotherapy and transmission control
IDM: Intensified disease management
MDA: Mass drug administration
MMDP: Morbidity management and disability prevention
NGDO: Non-governmental development organization
MoH: Ministry of Health
NNN: NTD Non-Governmental Organisation Network
